# Assessment IS learning: developing a student‐centred approach for assessment in Higher Education

**DOI:** 10.1002/2211-5463.13921

**Published:** 2024-11-01

**Authors:** Stephen Rutherford, Connie Pritchard, Nigel Francis

**Affiliations:** ^1^ School of Biosciences Cardiff University UK

**Keywords:** artificial intelligence, assessment, EAT framework, feedback, self‐regulated learning, student‐centred

## Abstract

Assessment and the associated feedback from those assessments are powerful factors in the development of students' learning. We have seen a shift within the Higher Education sector to conceptualise assessment as being more than summative assessment ‘of’ learning. Instead, there has been a greater emphasis on assessment ‘as’ learning, or assessment ‘for’ learning, through the enhanced use of formative assessments. Centralising assessment within the learning process highlights that assessment IS learning and cannot be separated from other elements of the learning process. In particular, assessment has a vital role to play in the development of students' self‐regulated learning skills and the development of independence in learners. However, for assessments to effectively support learning, they need to be meaningful, engaging, well‐integrated into the learning activities and ‘student‐focused’. Placing student skills development and personal development at the centre of assessment design has the potential to empower students through assessment. This review focuses on the potential of assessment to support student learning and development, using the ‘Equity, Agency, Transparency’ (‘EAT’) framework as a lens for effective assessment and feedback practices. We suggest ways in which we can make our assessment and feedback practices more inclusive, meaningful and authentic to the students' learning needs.

## Graphical abstract

Assessment IS learning, either assessment of learning, assessment for learning, or assessment as learning, or combinations thereof. Good assessment design, literacy and feedback underpin effective student‐centred assessment. The learner is central to a cyclical process involving the development of competences, acting on feedback received, self‐reflection on progress, and the development of self‐regulatory skills; leading to a capable graduate lifelong learner.

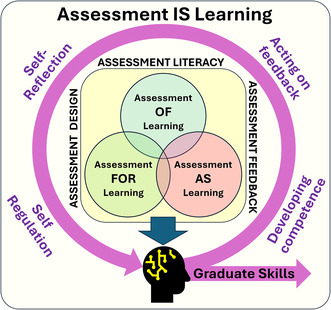

**Abbreviations**
AD: assessment design
AF: assessment feedback
AL: assessment literacy
EAT: equity, agency and transparency
GenAI: generative artificial intelligence
HE: higher education
SRL: self‐regulated learning

Assessment and feedback practices in Higher Education (HE) have been the subject of intense scrutiny and innovation for several years. In particular, attention has been paid to rethinking the role of assessment within the learning process [1, 2], the responsibilities of students and educators in assessment [3], and effective feedback practices to support student learning and development [3, 4]. Recently, paradigm‐shifting disruptions have forced radical changes in concepts of assessment, such as the COVID‐19 pandemic forcing widespread adoption of online and open‐book assessments [5], and the use of digital resources and generative AI making remote written assessments vulnerable to academic misconduct [6, 7]. However, despite these disruptions, evaluation practices within the HE sector are still very much focused on more‐traditional notions of assessment, and while the landscape is changing, the pace of change is slow.

## What is the purpose of assessment?

Of key importance is determining the role assessment plays within educational practices. Samuelowitz and Bain proposed a spectrum between teacher‐focused and learner‐focused learning and assessment [8, 9]. Teacher‐focused assessment primarily benefits the educator—confirming the student has met learning outcomes, providing metrics of attainment for the student's transcript, auditing learning and achieving these with minimal workload. Learner‐focused assessment drives learning processes, supports the student self‐auditing their progress, and motivates and empowers the student.

There has been a strong movement across HE sectors towards broadening concepts of assessment [10] from purely assessment ‘of’ learning (summative assessment that evaluates attainment), to assessment ‘for’ learning (assessment that supports and drives learning and enables the student to benchmark their progress; [11]) and assessment ‘as’ learning (assessments that deliver part of the curriculum, and/or equip students to develop understandings of themselves; [12]). A key paradigm shift, therefore, is the concept that assessment ‘is’ learning, and is integral to the student's ongoing development and integration within a discipline or community of practice, or as a citizen in a global community.

Boud [13] conceptualises three broad assessment purposes: assessments that assure (summative assessment measuring attainment; assessment of learning); enable (formative assessment; for/as learning); and build sustainability (developing self‐evaluation and lifelong learning skills; self‐assessment). In HE, students are supported intensively in their learning through scaffolded learning activities and assessments set by educators [14]. However, these activities should also build lifelong learning skills required post‐graduation. Boud [13] highlights that summative assessment needs to be low‐stakes initially, then increase in prominence through the course. Formative assessment should feature strongly at the start, but then gradually reduce as the student becomes more independent (formative assessment may inhibit independence by creating dependency on educators) [14]. Self‐assessment should be prominent throughout the learning journey, from the moment students begin their HE studies [13].

These categories of assessment are not exclusive, and assessment roles can overlap. For example, summative assessments can have formative and sustainable impacts, given appropriate guidance and feedback. What is key is that we consider the impact that assessment can have on the student as a learner, their ongoing learning journey and their development as a ‘self‐regulated learner’ [15, 16].

## Self‐regulated learning

**Fig. 1:**
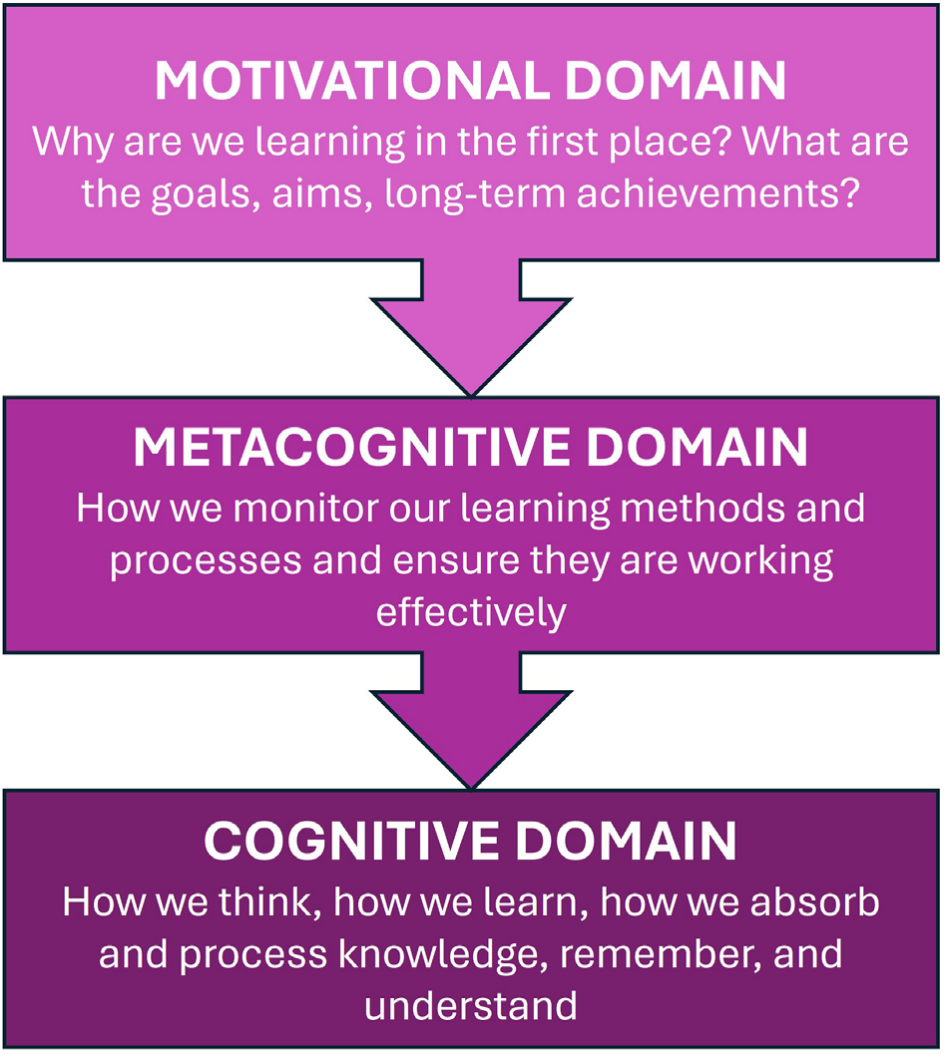
Self‐regulation domains. Models of self‐regulation focus on three ‘domains’, Cognitive, Metacognitive and Motivational/Affective. The metacognitive domain impacts the cognitive domain, and the motivational domain impacts both [20].

Key to becoming an independent learner in HE is the ability to self‐regulate one's learning [17, 18, 19]. Self‐regulation (SRL [20]) is a highly complex and nuanced area of educational research, including regulation of one's cognitive strategies, as well as behaviours, motivations and environments. Models of self‐regulated learning that focus on cognitive strategies, typically highlight three domains (summarised in Fig. 1): cognitive, metacognitive and motivational/affective [19, 20, 21, 22, 23]. The cognitive domain addresses how we learn and take on knowledge, skills and understanding; how we process information, retain it and retrieve it when required. The metacognitive domain focuses on the auditing and regulation of the cognitive domain, and how; learners evaluate the efficacy of learning strategies and schema. The motivational/affective domain focuses on motivations and rationales for studying, and our intended outcomes and goals. Additionally, Lehmann *et al*. [22] emphasise that each domain consists of two components: behaviours/actions and mental processes, each of which needs to be mastered by the learner.

For assessment to be effective as an instrument for learning, it needs to support the development of SRL. While SRL is primarily a personal process, interactions with others and the local environment are also important [24, 25, 26]. For example, co‐regulated learning (interactions between a learner and a more‐experienced mentor or teacher [20, 27]), and socially‐shared regulation (learning between peers [27, 28]), also have substantial impact. Assessment can provide avenues for all these forms of regulation. Panadero *et al*. [20] highlight that students require support to become reflective on their own capabilities through interactions with educators, resources and peers. In particular, guidance is required in order to transform external measurements of performance (marking criteria and standards) into internalised personalised standards, through which learners can benchmark their own performance. Therefore, explicit modelling of self‐assessment strategies should be embedded within the learning process [29]. Assessment needs to actively engage students both as learners and agents of their own development.

## The ‘Equity, Agency & Transparency’ (‘EAT’) framework—A research‐informed model for effective assessment

**Fig. 2:**
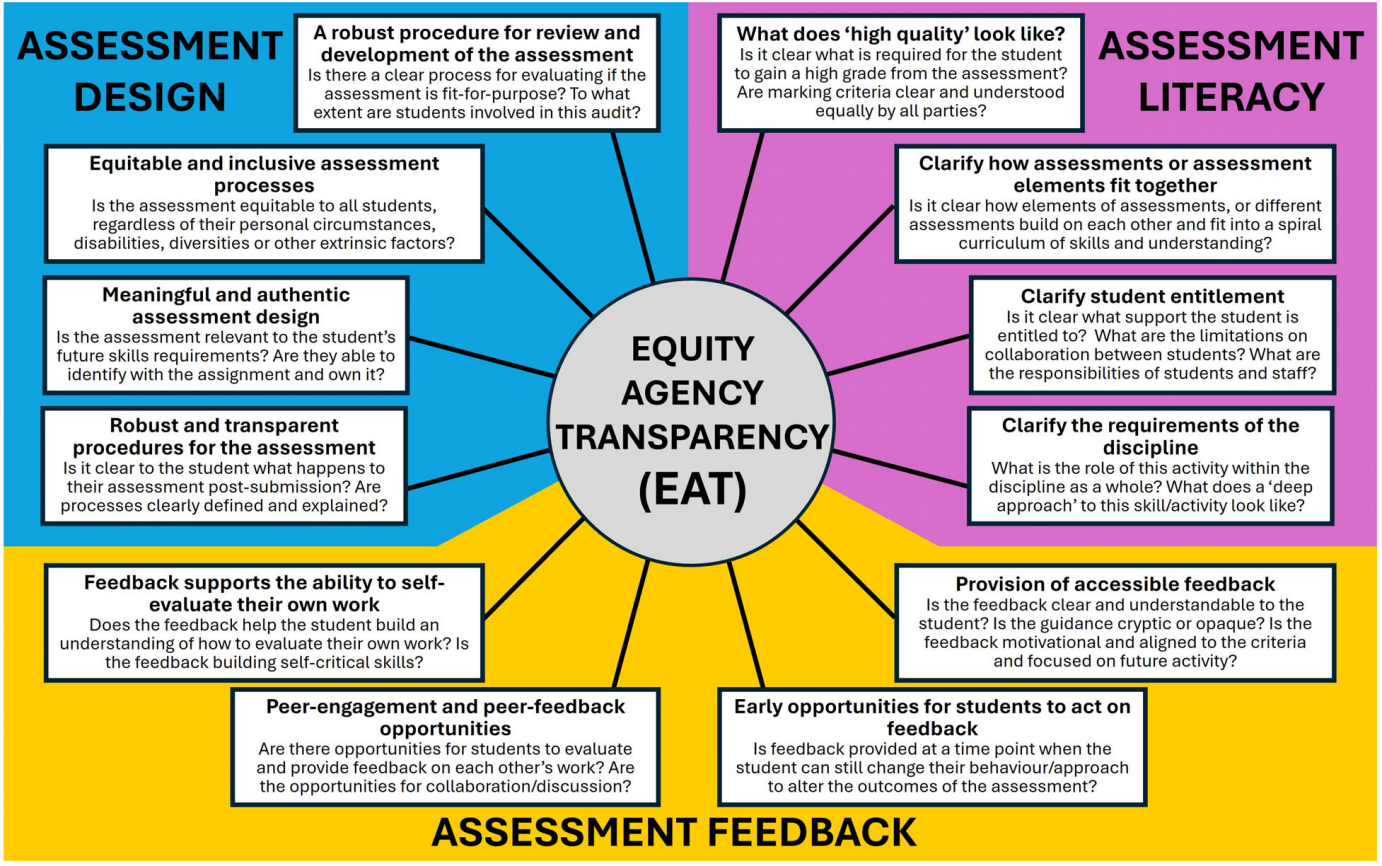
The Equity, Agency and Transparency (EAT) framework. The EAT framework [30, 31, 32] is subdivided into three dimensions: Assessment Design (blue shading), Assessment Literacy (purple shading) and Assessment Feedback (yellow shading). Each of these comprises of four subdimensions (smaller boxes). The subdimensions described here are focused around the ‘lecturer’ perspective of an effective assessment. Other perspectives of the framework focus on use by undergraduate and postgraduate students, and each have subtly different wording [30]. All subdimensions are linked, to indicate the intersectionality of the facets of assessment practice.

A powerful framework for evaluating the efficacy of an assessment and the extent to which it is focused on student learning is the ‘Equity, Agency and Transparency’ (‘EAT’) framework [30]. This research‐based framework, drawn from an extensive review of many thousand published studies on assessment, focuses the elements of effective assessment into three dimensions, each with four subdimensions (summarised in Fig. 2): Assessment Design (designing assessments that are robust, equitable, engaging and transparent); Assessment Literacy (understanding the parameters of assessment, and what impact an assessment can have on learning); and Assessment Feedback (designing feedback practices that support ongoing learner development). More details of applying the framework are summarised elsewhere [16, 31, 32]. For a more detailed exposition of SRL in assessment, see Evans [33]. EAT may be used to facilitate reflection on individual assessments or assessment practices at the course, departmental or institutional level. This review will utilise selected subdimensions of EAT to illustrate effective assessment practices to support SRL in HE. Within the limited scope of this review, it is not possible to address all 12 subdimensions/However, we have selected those which are the most relevant to assessment in biosciences or with the most easily achieved impact on making assessments more ‘student‐centred’. For each subdimension discussed, examples of potential applications to the biosciences are included.

## Rethinking assessment in the biosciences from an EAT perspective

### Assessment Design

Effective learning through assessment requires that assessment design to be optimal. The EAT Assessment Design subdimensions focus on elements that ensure effective and robust design for assessments, two subdomains are investigated below.

#### Inclusive assessment

Fundamental to all fair, robust and effective assessment practice is the need for all assessments to be inclusive and equitable for all learners [34]. Are we assured that all students have an equal potential to perform to their fullest in the assessment, regardless of their personal circumstances? Students with disabilities, neurodiversities, or from different cultures and/or are studying in their non‐native language, will face additional challenges compared to their peers when faced with specific assessment types or modalities. Representation of students within a teaching and assessment environment is also an important factor. There is substantial evidence for, and justified concern over, ethnicity awarding gaps in many HE sectors [35]. Whereby students from under‐represented ethnicities within the course or discipline, on average, have poorer academic outcomes than better‐represented ethnicities. In the UK, for Biological Sciences in 2019/2020, the proportion of students gaining a higher class (first or upper second) degree was between 9.8% and 21.4% lower for non‐white students compared to white students [36]. Other areas of concern are digital poverty [37], and time poverty [38, 39], both of which disproportionately impact students from disadvantaged socioeconomic backgrounds. These students will face additional challenges to more affluent students in completing assignments.

Engaging with concepts promoting equity in assessment, such as Universal Design for Learning (UDL) [40, 41] principles can be a major assistance in ensuring inclusive practice. UDL highlights three principles to making assessments inclusive (adapted here to relate to assessment). (a) Engagement: designing optionality in the assessment to welcome students of different identities, capacities, persistence levels and emotional capacity. (b) Representation: designing in language, examples, symbols and knowledge that will be relatable for all diversities of students. (c) Actions and expression: designing optionality into the assessment process (assessment type, timing, focus or length), to accommodate the most common diversities in the student group. Designing an assessment to be flexible enough to easily accommodate the most common diversities in a class benefits all learners in that space, not just those with specific needs [42]. Providing elements of student choice within an assessment can be a powerful agent for inclusion [43], either in the subject matter of an assignment (addressing representation issues) or the mode of assessment (addressing many accessibility issues). However, Tai *et al*. [43] also argue that we need to rethink all aspects of assessment: the subject matter, modality, purpose, timing and procedures of our assessments; to build‐in accommodations needed for students with additional needs or personal challenges.

Applying these principles for assessment in the biosciences is best approached by considering what the most common diversities, disabilities or challenges the particular student group might have, then balancing these against fundamental skill requirements of a bioscientist. Where there are absolutely essential skills or competencies to evaluate, then support needs to be provided to students with additional needs. Where the assessment aims do not specifically include a particular performative skill, a simple approach to increase inclusivity is to build‐in options in how the assessment can be undertaken—for example, choosing a written output or a presentation. Students with disabilities of the written word would therefore have a viable alternative, while those who have challenges with confidence or anxiety would also be accommodated. Most of the marking criteria (e.g. critical thinking, use of evidence, core knowledge) can remain the same for either modality, but with criteria for presentation being set separately for each mode of delivery. That therefore supports consistency, but provides student with a choice of using a format with which they are more comfortable.

#### Meaningful/authentic assessment

An important Assessment Design subdimension for bioscientists is designing assessments that are meaningful to the student. In order for a student to understand the rationale for an assessment, it helps to mimic or parallel actual actions and performative skills the student will need in their working life. This alignment of assessment with real, functional, practical skills and competencies is often termed ‘Authentic Assessment’ in the literature [44, 45, 46], although that term is considered controversial or pejorative by some.

There are several assessment modalities used in HE which perform useful functions of testing knowledge (e.g. multiple choice tests), critical analysis and argumentation (e.g. essays) and minimising potential for academic misconduct (e.g. invigilated, time‐limited examinations). However, these activities are unique to the educational environment and often require the development of skills and ‘exam technique’ that is of little or no use to the student outside of university. Making assessments more authentic to the skills of the discipline enhances assessment ‘as’ learning. The student develops important graduate competencies through the authoring/performance of their assessment.

Evidence suggests that assessments aligned to discipline‐specific skills and activities create meaningful learning environments for the student [46, 47, 48], reduce cognitive load [49] and foster greater engagement and ownership of the activity [50]. This parallels with the concept of meaningful assessments for a student [51], whereby the student is able to connect personally with the assessment as being directly relevant to their own interests, goals, ambitions, expectations or experiences. A student who engages meaningfully with an assessment is far more likely to be able to internalise content, apply it externally, and apply substantial effort [45], and will be far less likely to want to cheat or take shortcuts to the output [52].

Examples of authentic/real‐world assessment types for Biological Sciences are the writing of a scientific paper or report, the development (or outlining) of a grant proposal to investigate a subject of interest; or a short presentation on a scientific subject. More‐abstract assessments, such as an essay, could, for example, be re‐framed as a ‘position‐paper’ to a biotech company or government organisation. Thus, providing a balanced and critical overview of a subject, but with a real‐life output in mind. Multiple choice tests may be better framed around problem‐solving questions or evaluating data; activities which require baseline knowledge, but are using that knowledge in a scientific context, rather than just factual recall. Contextualising knowledge aids in retention [53, 54] and so such assessments would also be more impactful on student learning.

Making assessment more‐meaningful could be as simple as enabling students to choose the subject of focus for the assessment, thus aligning with their own interests. A potential approach could be to partner with a local Biotech company and identify from them a real problem they face that needs to be solved. This then becomes the assessment brief for the students, who need to understand the subject and the problem, before they can determine a solution. The students would then would be working on a real issue, with potentially real impact, while gaining an insight on industry. The partner company would also benefit through being provided with a range of potentially useful student‐authored solutions to their problem. Meaningful assessment does more than just assess knowledge or competence, it is also an opportunity to teach the student about the discipline, and to embed them in a wider community of practice [55] of the discipline as a whole.

### Assessment Literacy

The EAT Assessment Literacy subdimensions ensure that there is a full understanding of what assessment is, and the potential support it can provide for the learner and/or educator.

#### Criteria and standards

The first Assessment Literacy subdimension focuses on ensuring that all parties have a clear (and shared) understanding of what a high‐quality output looks like. Are there marking criteria for the assessment? Are the students aware of the criteria, and do they understand them? [56, 57]? A key element here is ensuring that the students are able to internalise those standards and develop an intuitive understanding of what constitutes a high‐quality output. This understanding can be challenging for two major reasons: Firstly, the language of marking criteria can be somewhat arcane to students, involving terms with which they may not previously have experienced (e.g. ‘Critical Analysis’, ‘effective structure’, ‘cohesive argument’). Unfamiliar terms need to be explained to students. Secondly, descriptors of successive grade levels can be unhelpful [58, 59], with the same descriptors being used, prefaced only with vague qualifiers (e.g. ‘Satisfactory’, ‘Good’, ‘Excellent’) to indicate different levels of expectation. These terms have no clear quantitative meaning to a student and can be difficult for them to conceptualise and apply. Actively engaging students in discussing marking criteria (e.g. a 5‐min discussion of one criterion element each, in successive classes [60]), is a good way to highlight that the criteria exist, what they mean, and how to apply them. This approach could easily be used for Bioscience students in a large lecture context, as a ‘mid‐lecture break’ activity, to help with resetting students' attention‐span on the lecture [61, 62] as well as supporting them in understanding expected standards.

Another key challenge beyond understanding the language of marking criteria is ensuring that students have the same interpretation of expected standards of those criteria as their educators and that educators have the same interpretations as each other. Differential interpretations of weighting of elements of the criteria between markers are problematic [15, 63]. There is plentiful evidence to show that different markers evaluating the same work using the same criteria, will assign grades based on their own personal biases for the importance of different elements of the criteria [56, 64, 65]. This is a particular challenge in the Biosciences, where criteria typically involve judging content knowledge, analytical ability, clarity/structure and the ability to use up‐to‐date evidence. Key to addressing this is veering away from holistic marking criteria and using rubrics [66] where each criterion element is separated out and given a pre‐agreed weighting [67, 68, 69]. The marker, therefore, only provides a value judgement for each element of the rubric. Another way of addressing standardisation is social moderation of marking [64], where multiple markers of a single assessment grade initially the same small handful submissions, then discuss their marks with each other before marking the remaining allocated scripts.

#### Alignment to the discipline

**Fig. 3:**
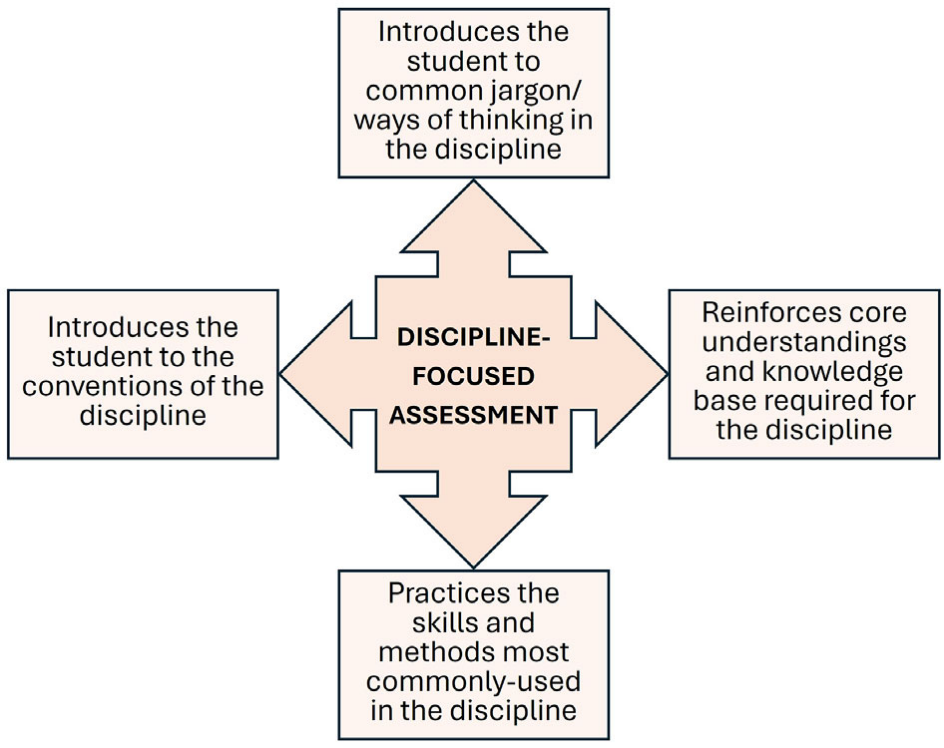
The potential impacts of discipline‐focused assessment. Affordances from discipline‐focused assessment. Disciplinary focus enhances student engagement with the core knowledge, conventions, discourses and skills that underpin the discipline.

Another key Assessment Literacy subdimension, which ties in closely with meaningful/authentic assessment (discussed later), is clarifying the relevance of the assessment to the discipline [70]. Is it clear to the students how the assessment is relevant to their own development and the requirements of the biosciences? Assessment can positively impact by introducing a student to the culture, norms and ways of thinking within a discipline [71] (summarised in Fig. 3). Aligning the assessment with the requirements of the discipline embeds discipline‐specific skills and methods, reinforces core disciplinary knowledge and introduces the student to the conventions of the discipline and to its discourse and jargon. Within the Biosciences this could involve framing an assessment within the context of a series of scientific experiments, or by reviewing the work of a pioneering scientist or a seminal research paper. Contextualising the assessment within the actual activities of the discipline provides opportunities to introduce students to the ways of working of a bioscientist—such as problem‐solving in the laboratory, interpreting and presenting data, designing experiments or presenting findings using the conventions of peer‐reviewed journals. A simple example of this is to require students to format a report according to the Author Guidelines of a named journal. This requires them to research those guidelines and see them used within a real context.

Also fundamental to this subdimension is being clear what a ‘deep’ engagement with the discipline looks like. Entwhistle *et al*. [72, 73, 74] classify learning approaches as either surface (memorisation, repetition, with limited intellectual engagement) or deep (understanding core principles, integrating concepts together). So what does a ‘deep’ engagement with the Biosciences look like for a student? It is important to clarify core concepts (fundamental understanding/skills) and threshold concepts (enable a learner to reshape their thinking to align with the discipline [75]) and how these underpin the discipline as a whole. Assessment can help differentiate between these concepts. For Biochemistry, for example, what are the concepts that a biomolecular scientist requires intuitively, or those required in order to apply knowledge to practical scientific inquiry?

### Assessment Feedback

Developing student and staff feedback literacy is of fundamental importance [76]. The Assessment Feedback subdimensions focus on ensuring that feedback is of clear use to the student in developing their ability to evaluate their work and provide self‐feedback on their progress/outputs. The engagement and use of feedback are a specific learning activity in itself, aligned to, but not subsumed by, undertaking assessment [77].

#### Clear and timely feedback

The first two Assessment Feedback subdimensions highlight the importance of providing feedback that is clear, understandable and unambiguous to the student [2, 78] and provided at a timepoint when it actively supports the student in their performance [2, 79]. The majority of feedback practices within the sector focus on feedback on final submissions of work [80]. However, this feedback is often unclear to the student (with cryptic comments such as ‘Good’, ‘No!’, ‘Referencing!’ and ‘Excellent’ being meaningless without additional context) or focused overmuch on activities the student did, without also highlighting clearly how to apply that to future work. Conversely, feedback provided while the student is still developing their output for the assessment/assignment empowers them to change their approach and behaviour to alter their final outcome and embeds that learning actively. Feedback therefore acts as a change agent for the student, enhancing their SRL and self‐evaluation skills. Even future‐focused feedback on a final assessment can be limited in its impact, due to the student misremembering the guidance when used for a later assignment [81].

Feedback is also typically didactic and one‐way, rather than dialogic and providing the student the opportunity to ask questions [80, 82]. Limiting the student's opportunities to discuss their feedback limits their learning gain [83]. Feedback is not equally useful to all students, as each student is an individual with personalised approaches to learning. For example, Orsmond and Merry [84] identified that bioscience students of higher or lower attainment levels engage differentially with different forms of feedback (lower‐attaining student preferring specific, directive feedback, high‐attaining student preferring more generic, conceptual feedback). Therefore, personalised approaches to feedback, that are tailored towards the needs, goals and motivations of the student are important.

One of the most powerful forms of feedback, and one which is of particular benefit in the Biosciences, is audio [85] or video [86] feedback. Most people can speak faster than they can write or type, and so it is possible to provide detailed and well‐explained feedback by annotating students' work, aligned to points made in an audio file or screen capture. By recording these in the moment, as the work is graded, this approach is also rapid for the marker, as well as more explicit to the student. The additional benefit of this approach is that the feedback feels more personalised, and the tone of voice provides more nuance to the comments, and can soften the negative impact of criticisms, so that the all‐too‐common negative emotional response to feedback [87] is avoided.

#### Facilitating self‐evaluation

It is essential to empower students to operate as ‘judges of their own learning’ [88], and we must ensure that there are opportunities for self‐assessment and self‐feedback built into courses [89, 90]. Self‐assessment is fundamental to the self‐regulation of learning [91] and empowers the student to internalise the required standards that we, as educators, set [92]. Another Feedback subdimension encourages us to question whether our feedback practices support the student in developing this essential skill of self‐evaluation and self‐feedback [93]. Is the student able to see through our eyes as they engage with their feedback, and understand the thought processes we went through while marking their work? Are we guiding the student to be self‐reflective as we shape feedback? Carless's work on self‐evaluation [94] and self‐generated feedback [95] is particularly powerful here, focusing on methods of encouraging students to meaningfully self‐evaluate their work, developing strategies for change. Sadler [96] emphasises the importance of supporting students to self‐evaluate their progress and approaches while they are in the process of performing the assessment activities.

**Fig. 4:**
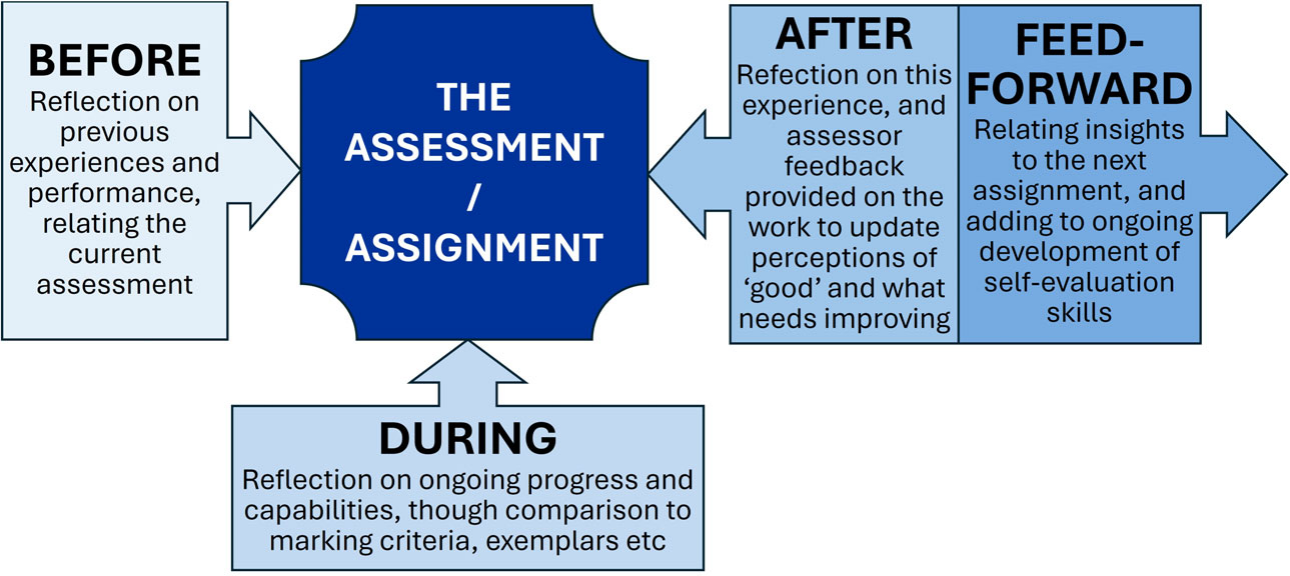
Self‐feedback activities at stages of the assessment journey. The boxes each show a stage in the time course of an assessment (before and during the assessment, immediately after submission and upon receiving feedback). Potential self‐reflective uses for the feedback are indicated.

Self‐feedback has impact throughout the assessment process (summarised in Fig. 4). Developing student feedback literacy is essential for this impact to be felt [97]. Before the assessment, reflection on previous experiences and former feedback empowers the student to adopt new approaches. During the assessment, self‐assessment can ensure alignment with the criteria and staying focused on the assessment aims. After submission, while the assessment is fresh in the mind, reflection on what worked well and what skills needed improvement is valuable. After receipt of the grade and marker feedback, self‐evaluation compares one's own perceptions with those of the marker and helps refine and reinforce internal quality standards [95].

Self‐feedback can also be used to ensure students follow guidance. For example, for an assessment as a scientific report, students could be required to complete a self‐assessment proforma, where they rank their adherence to key requirements of an Introduction, Methods, Results or Discussion section. The students therefore reflect on their performance, but the proforma also reiterates what the assessment guidelines expect to be included, and can serve as a prompt to students who have not included some of these. Requiring student to self‐reflect on aspects of an activity as part of an assessment is also a powerful tool for self‐development and learning.

#### Peer evaluation and feedback

**Fig. 5:**
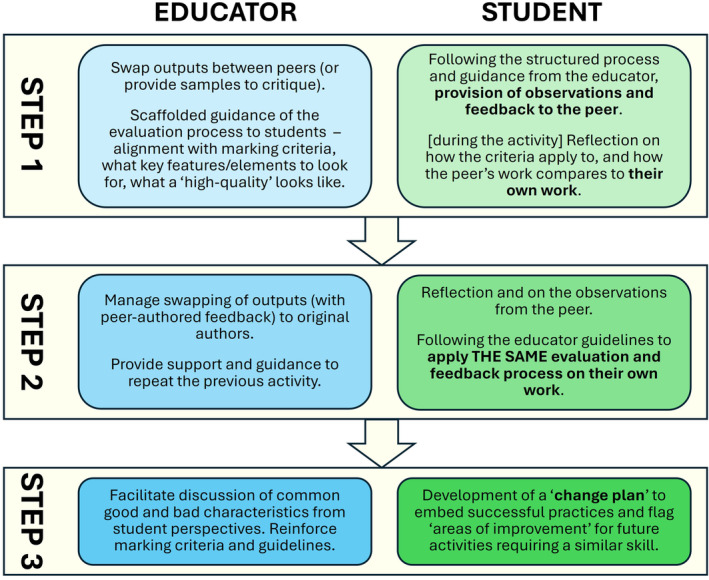
Three‐stage model of educator‐facilitated peer feedback. The educator (blue shading) directs scaffolded activities for the learner (green shading) to undertake. The latter stages involve reflection on the peer assessor's own work, the fundamental aim of the exercise. The educator needs to facilitate the surfacing of this self‐reflection.

A powerful agent in developing self‐evaluation skills is peer evaluation [98, 99], the final Assessment Feedback subdimension. Peer evaluation is often adopted in a sub‐optimal way, which students find disconcerting and disengaging [100, 101]. The act of evaluating another can be seen as either useless (a non‐expert assessing a piece of work), daunting or unfair and doing the educator's job for them. Instead, the focus needs to be not on the product of the peer assessment, but on the peer assessor and their learning [102, 103]. The process of peer evaluation and peer feedback needs to be a scaffolded process (illustrated in Fig. 5), whereby, initially, the peer assessor is guided through the evaluation process by the educator, providing feedback on a peer's work [89]. The educator can use this activity to train the peer assessor to appreciate and internalise required standards [57, 103]. The second stage, which often is omitted, is the most important; to have the peer assessor then replicate the evaluation/feedback process on their own work [102]. Finally, each student builds a change plan, to note important points to remember in future. Through this three‐stage process, the peer assessor develops their self‐evaluation abilities. The mark and/or feedback received from a peer assessor has less impact on student learning gain than the act of giving feedback to another [104].

Peer feedback is used effectively by the authors of this review as a means of teaching scientific writing skills to Year 1 students, following the process in Fig. 5. For each of five modules/courses in Year 1, students are required to write either a set of figures or an introduction, methods, results or Discussion section. Guidance for each activity is given via video recordings when each assessment is set. Students' outputs are then brought as hardcopies to a facilitated peer feedback session, where the students are guided through reviewing a peer's work. They are then guided through the process again on their own work. They note down key learning points from the session, and these reflections are then used to guide them in a final assessment, which is a full scientific paper report. The students therefore gain two experiences of reviewing an exemplar of work (including their own) and take the feedback forward to the next assessment.

Peer evaluation need not be undertaken on a peer's work (evaluation of a colleague can be uncomfortable for students [100]). Instead, evaluation could be of an exemplar [105], a previous student's work (with permission of the author), or even on an output generated by Generative Artificial Intelligence (GenAI). The scaffolded act of the student providing feedback on an artefact is the key to the learning gain; however, it has more authenticity if there is a real recipient for that feedback. This activity is arguably the most important and impactful of any learning activity in which the student might engage. The process does not have to involve providing a grade [57]; it is the guided reflection on the reviewing process that is powerful for learning.

This last comment highlights one element which is currently a major issue within the assessment activities in HE, the use of GenAI. While this technology has the potential to be problematic in student assessment processes, through academic misconduct, it does have potential benefits for the development of SRL through assessment.

### The role of GenAI in enhancing student self‐regulation through assessment

Integrating GenAI into assessment and feedback practices offers the potential to enhance self‐regulated learning (SRL). Here are only brief suggestions of how GenAI can support student‐centred assessment. For a more in‐depth discussion of GenAI across all dimensions of the EAT framework, please see Evans and Waring [16]. For more general guidance on GenAI in assessment, see guidance by Francis and Smith [106].

#### Personalised feedback

Aligned with the principles of clear and timely feedback, GenAI can provide real‐time feedback to students, engaging them actively in the feedback process as they are creating the assessment output. This process may help guide the students on structure, clarity or language use, which in itself is a learning experience for the student, provided that they quality‐check the GenAI output. This is an evolution of standard tools such as spelling and grammar checkers that exist in all word‐processing packages. GenAI‐powered tools can instantaneously assess student performance, offering guidance and corrections that students can apply immediately. This immediacy potentially helps students internalise the feedback, understand their mistakes and learn how to avoid them in the future, thus enhancing feedback for learning [15] and, with proper guidance, supporting self‐evaluation approaches.

Moreover, GenAI tools can offer feedback on drafts, allowing students to revise their work before final submission. This iterative process provides multiple opportunities for feedback, helping close the feedback loop and ensure continuous improvement. GenAI can deliver highly individualised feedback tailored to each student's specific needs at the precise point it is needed in their learning journey. This immediate, personalised feedback helps students understand and address their weaknesses, thus promoting continuous improvement and deeper learning [107]. This is an approach undertaken by many within the Bioscience sector (in industry and academia) and therefore is aligning the students with a key employability skill on graduation.

The capacity of GenAI to offer personalised feedback supports students in critically engaging with their learning tasks. Analysis of individual learning patterns can provide customised learning opportunities, fostering a more engaging and effective learning experience [88]. GenAI platforms can also help students set realistic and achievable goals, enabling them to manage their learning journeys actively. The continuous, collaborative nature of GenAI‐driven learning platforms promotes sustained engagement and self‐regulation from students [97].

#### Meaningful assessment

GenAI can transform assessments into learning opportunities by presenting students with working‐world problems that require the application of their knowledge and skills. This authentic approach makes learning more relevant and meaningful, encouraging students to engage deeply with the material [20]. GenAI systems can facilitate student‐centred learning by adapting to individual needs and providing support and resources tailored to each student. Furthermore, GenAI can support evidence‐based practices by analysing vast amounts of data to identify the most effective teaching and assessment strategies, ensuring that educational practices are continuously improved and updated [103].

The continuous evaluation of GenAI tools is essential for maintaining their effectiveness and relevance. GenAI can provide data‐driven insights into student performance and learning behaviours, allowing educators to make informed decisions about instructional strategies and assessment designs [108]. GenAI systems can adapt assessments in real time, responding to the immediate needs of students and ensuring that assessments remain challenging yet attainable. Through GenAI, quality assurance processes can be enhanced by ensuring that assessments are fair, reliable and valid by continuously monitoring and adjusting assessment practices based on data‐driven insights [109].

## Conclusions

Assessment and feedback are fundamental to the learning journey of the student. Designing assessment activities and feedback opportunities into our teaching practices is key to supporting our students in developing self‐regulatory skills. Utilising assessment as a learning tool, recognising that assessment ‘is’ learning, and adopting the affordances of technology to support this, has the potential to enhance our students as independent learners and to be agentic in their own learning.

Within the bioscience sector, assessments are often highly focused on testing content and understanding. While these are essential to underpin scientific inquiry, we also need to consider supporting students in the application of this knowledge to key scientific skills of problem‐solving, communication and our responsibilities as scientists within society. All of these are factors that can and should be included in assessment of 21st‐Century science students, regardless of their eventual occupations. Enhancing assessment that builds students' skills, as well as testing their competence, is a powerful means of achieving this aim. Rethinking our curricula from a perspective that assessment is learning should enable us to build‐in assessment as a learning tool throughout the student's learning journey.

## Conflict of interest

The authors declare no conflict of interest.

## Author contributions

SR and NF conceived the article, and all authors contributed equally to the research and authoring of the article.
